# Lack of Methylene Blue Staining in Superficial Epithelia as a Possible Marker for Superficial Lateral Spread of Bile Duct Cancer

**DOI:** 10.1155/DTE.3.29

**Published:** 1996

**Authors:** I. Maetani, S. Ogawa, M. Sato, Y. Igarashi, Y. Sakai, K. Shibuya

**Affiliations:** 1 Division of Digestive Endoscopy Toho University Ohashi Hospital Tokyo Japan; 2 Department of Pathology Toho University Ohashi Hospital Tokyo Japan; 3 Third Department of Internal Medicine Toho University School of Medicine Tokyo Japan; 4 2-17-6 Ohashi Meguro-ku Tokyo 153 Japan

## Abstract

Longitudinal cancer spread is very important for staging of resectability in bile duct cancer. We verified the difference in methylene blue staining properties between cancerous and noncancerous epithelia that are usually observed by cholangioscopy. We obtained 45 biopsy specimens from the common bile duct of 20 patients with bile duct disease using percutaneous transhepatic cholangioscopy (PTCS) after staining with 0.05% methylene blue. We compared the microscopic staining properties with the gross endoscopic observations and evaluated the characteristics of methylene blue staining on frozen sections of each type of cholangial epithelium. Microscopic staining properties were significantly associated with endoscopic observations (*p* = 0.00001). While 18 of 20 (90%) specimens of normal epithelia stained with methylene blue, 11 of 16 (69%) specimens of metaplastic epithelia were stained, with no staining obtained in cancerous epithelia. The cancerous epithelia stained significantly less often than either the normal (*p* = 0.000005) or the metaplastic (*p* = 0.001) epithelia. Evaluation of methylene blue staining during PTCS revealed that this stain was absorbed by the cholangial epithelia, not superficially stuck to it. The difference in methylene blue staining properties between the cancerous and normal epithelia could be helpful to clarify the boundary of superficial lateral spread of bile duct cancer.


*Diagnostic and Therapeutic Endoscopy*, Vol. 3, pp. 29–34
Reprints available directly from the publisher
Photocopying permitted by license only

© 1996 OPA (Overseas Publishers Association)
Amsterdam B.V. Published in The Netherlands
by Harwood Academic Publishers GmbH
Printed in Singapore

### Lack of Methylene Blue Staining in Superficial Epithelia as a Possible Marker for Superficial Lateral Spread of Bile Duct Cancer

I. MAETANI*,†, S. OGAWA, M. SATO, Y. IGARASHI, Y. SAKAI and K. SHIBUYA

*Division of Digestive Endoscopy (I.M., Y.S.) and Department of Pathology (K.S.), Toho University Ohashi Hospital, and Third Department of Internal Medicine (S.O., M.S., Y.I.), Toho University School of Medicine, Tokyo, Japan*

*(Received 28 November 1995; In final form 29 February 1996)*

**Longitudinal cancer spread is very important for staging of resectability in bile duct cancer. We verified the difference in methylene blue staining properties between cancerous and noncancerous epithelia that are usually observed by cholangioscopy. We obtained 45 biopsy specimens from the common bile duct of 20 patients with bile duct disease using percutaneous transhepatic cholangioscopy (PTCS) after staining with 0.05% methylene blue. We compared the microscopic staining properties with the gross endoscopic observations and evaluated the characteristics of methylene blue staining on frozen sections of each type of cholangial epithelium. Microscopic staining properties were significantly associated with endoscopic observations ($p = 0.00001$). While 18 of 20 (90%) specimens of normal epithelia stained with methylene blue, 11 of 16 (69%) specimens of metaplastic epithelia were stained, with no staining obtained in cancerous epithelia. The cancerous epithelia stained significantly less often than either the normal ($p = 0.000005$) or the metaplastic ($p = 0.001$) epithelia. Evaluation of methylene blue staining during PTCS revealed that this stain was absorbed by the cholangial epithelia, not superficially stuck to it. The difference in methylene blue staining properties between the cancerous and normal epithelia could be helpful to clarify the boundary of superficial lateral spread of bile duct cancer.**

*Keywords:* Bile duct cancer, mucosal spread, percutaneous transhepatic cholangioscopy, methylene blue, staining property

### INTRODUCTION

Longitudinal cancer spread is very important for staging and assessment of resectability in bile duct cancer. Percutaneous transhepatic cholangioscopy (PTCS) is highly useful in the diagnosis of malignant biliary stenosis, especially of the mucosal spread of carcinoma in the bile duct. Since 1987, we have usually performed PTCS to accurately evaluate all patients with malignant-appearing bile duct strictures. A papil-

*Corresponding author. Tel.: 03-3468-1251. Fax: 03-3468-1269.
†Present address: 2-17-6 Ohashi Meguro-ku Tokyo, 153, Japan.

logranular surface of the bile duct reportedly indicates the presence of such mucosal spread [1–4]. However, this finding is reportedly related not to carcinoma, but rather to the papillary proliferation of the superficial epithelia [4]. Thus, the precise diagnosis of mucosal spread of biliary carcinoma may be difficult by only PTCS observation.

Methylene blue staining is reportedly useful for observing the mucosal surface and diagnosing lesions in the stomach and colon. We recently applied this staining method to the diagnosis of bile duct disorders [2,4]. We previously found that mucosa covered with non-neoplastic epithelium stained with methylene blue, whereas the mucosa covered with neoplastic epithelia did not stain under PTCS observation with an irrigating saline solution. To study these phenomena, we examined the staining properties of the bile duct epithelium by evaluating frozen sections of biopsy materials taken from the bile duct during PTCS, which was described by Tada *et al.* [9].

### MATERIALS AND METHODS

We studied 45 biopsy specimens taken from the common bile duct of 20 patients with biliary disease (Table I). There were 12 men and 8 women aged 31 to 88 years (mean 69.8 years). Before PTCS was conducted, percutaneous transhepatic cholangiodrainage and dilation of the sinus tract were performed up to 16 or 18 Fr. About 7 days later, we performed PTCS through the sinus tract without a sheath using a CHF-P20 or CHF-T20 cholangioscope (Olympus). At first, regular observation with saline was performed. Then, 20 ml of methylene blue solution (0.05%) was injected into the bile duct via the working channel of the cholangioscope. Three minutes later, this solution was aspirated, and the bile duct lumen was washed with saline solution using the scope. When no remaining methylene blue solution was observed in the bile duct lumen, we began our observation of the bile duct mucosa irrigated with saline.

TABLE I Clinical diagnoses of 17 patients from whom 39 biopsy specimens were obtained

| | No. of patients | No. of specimens |
|---|---|---|
| Pancreatic cancer | 7 | 17 |
| Common bile duct cancer | 6 | 12 |
| Gall bladder cancer | 2 | 5 |
| Common bile duct stone | 2 | 4 |
| Gastric cancer | 2 | 4 |
| Congenital biliary dilatation | 1 | 3 |
| Total | 20 | 45 |

We first determined whether the microscopic staining properties of the tissue were associated with the endoscopic characteristics recognized during PTCS. We then evaluated the staining characteristics of the superficial epithelia of the bile duct.

Some biopsy specimens were obtained with forceps during PTCS. After these biopsy specimens were frozen in OCT compound, two frozen sections were obtained from each specimen. One section was used as the nonstained section, while the other was stained with hematoxylin and eosin (HE). Non-HE stained sections were available for the examination of the staining properties of methylene blue with the results classified as stained and unstained (Figs. 1 and 2). We regarded stained epithelia of any degree being the stained group. HE-stained sections were available for the diagnosis of superficial epithelia: normal, metaplastic, and cancerous epithelia. Fisher's exact probability test was used for statistical analysis of the data. $p < 0.05$ was considered to be statistically significant.

### RESULTS

### Relationship between Endoscopic Methylene Blue Staining Characteristics and its Absorption by Cholangial Epithelia

Seven biopsy specimens that were taken from a region seen on PTCS to be stained, were not stained microscopically, including 2 normal epithelia, 2 metaplastic epithelia, and 3 cancerous epithelia (Table II). All but 7 specimens taken from the regions observed to be stained on PTCS showed staining microscopically. No stained materials were seen microscopically in the specimens taken from the regions observed to be unstained.

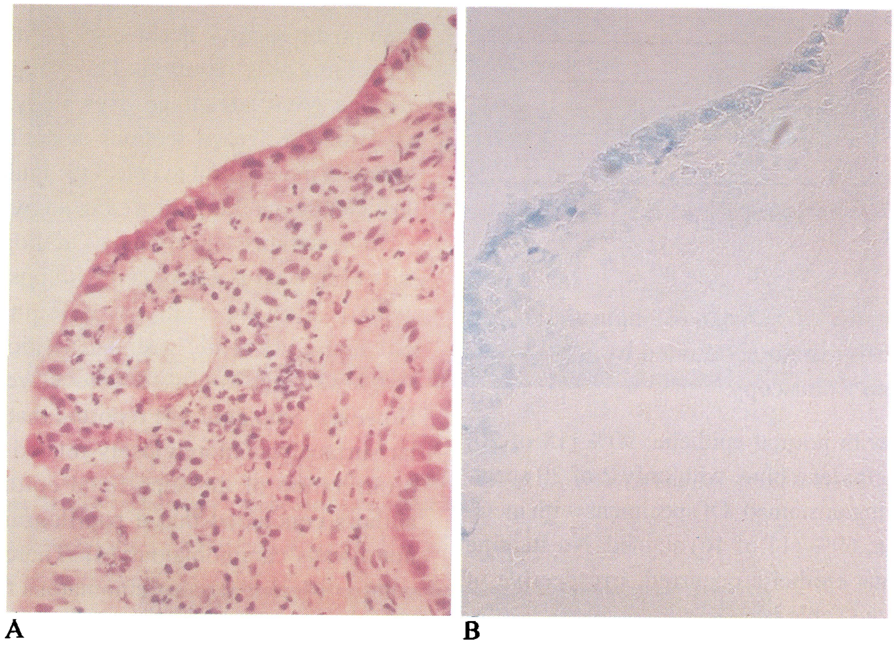

FIGURE 1 Micrographs of the cholangial normal epithelia stained with methylene blue (example of stained epithelia). A. Section stained with HE showing normal epithelia. B. Same section without HE showing epithelia to be well stained with methylene blue.

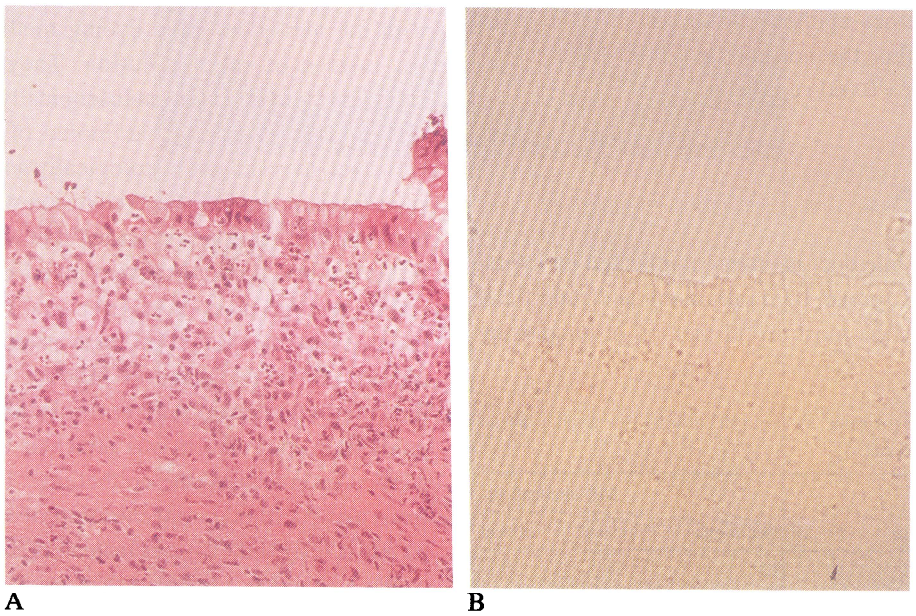

FIGURE 2 Micrographs of the cholangial metaplastic epithelia unstained with methylene blue (example of unstained epithelia). A. Section stained with HE showing normal epithelia. B. Same section without HE showing epithelia to be unstained with methylene blue.

TABLE II Comparison of the absorption characteristics of methylene blue for cholangial epithelia in the biopsy specimen vs. gross endoscopic observations in that region

| Endoscopy | No. of specimens | MB absorption present | MB absorption absent |
|---|---|---|---|
| Stained | 36 | 29 | 7 |
| Unstained | 9 | 0 | 9 |

*P = 0.00001 by Fisher's exact probability test
MB: methylene blue

### Staining Properties of Normal, Metaplastic, and Cancerous Epithelia Evaluated by Microscopy and Endoscopy

Of specimens with normal epithelia, 90% (18 of 20) stained with methylene blue, with only 2 of 20 specimens (10%) being unstained. Of specimens with metaplastic epithelia, 69% (11 of 16) stained. No staining of the cancerous epithelia occurred, irrespective of grade of staining (Table III).

Microscopic staining properties were associated with those observed endoscopically ($p = 0.00001$). Findings showed that most of the normal and metaplastic epithelia stained with methylene blue (Fig. 3), whereas the cancerous epithelia was unstained (Fig. 4). The cancerous epithelia stained significantly less often than either the normal ($p = 0.000005$) or the metaplastic ($p = 0.001$) epithelia.

### DISCUSSION

Cancer of the bile duct is often complicated by lateral carcinomatous spread, both intramural and superficial [5,6]. PTCS is the most useful method of diagnosing such lateral superficial spread precisely [1–4]. In some patients with cancer extension seen on PTCS before attempted resection, hepatectomy with pancreatoduodenectomy was required. This suggests that PTCS offers a great advantage over analysis of resection margins with frozen sections at the time of surgery. And, it is more useful to detect the multiple foci of bile duct carcinoma that followed in a few patients.

TABLE III Absorption of methylene blue by each type of cholangial epithelium

| Type of epithelium | No. of specimens | MB absorption present | MB absorption absent |
|---|---|---|---|
| Proper | 20 | 18 | 2* |
| Metaplastic | 16 | 11 | 5** |
| Cancerous | 9 | 0 | 9 |

*p = 0.000005, **p = 0.001 by Fisher's exact probability test
MB: methylene blue

Some authors report that a papillogranular surface of the bile duct is associated with positivity for carcinoma [1,3,4], especially with noninvasive mucosal carcinoma [4], while vascular dilation of cholangioscopic findings is related to invasive carcinoma [4]. However, Sato *et al.* [4] reported that a papillogranular surface was more closely related to the papillary proliferation of the superficial epithelia [4]. Those authors concluded that a papillogranular surface is not always carcinomatous [4]. Therefore, it is difficult to differentiate a carcinomatous from a noncarcinomatous papillogranular surface. In the stomach, carcinomatous epithelium was not stained with methylene blue; therefore, the methylene blue staining method could be helpful to clarify the boundary between cancerous epithelium and noncancerous epithelium [7]. Miyakawa *et al.* [8] reported PTCS with the methylene blue dyeing method using $CO_2$ gas instead of saline solution. They reported that cholangial mucosa was endoscopically dyed in 47% of lesions out of adenocarcinoma of the bile duct. However, they did not histologically ascertain that the superficial mucosa of the dyed lesion was adenocarcinoma or not. And they reported that all erosions and inflammatory granulomas were dyed intensely. Their results that the mucosa was changed to blue even in the area lacking epithelium, such as erosion and granuloma, can be explained as follows. They observed that methylene blue was not only absorbed in the epithelium, but also it was attached to the mucosal surface or mucus on the epithelium. This may result from using a higher concentration of methylene blue (0.5 to 1.0%) and $CO_2$ gas instead of saline solution.

We previously observed that areas of the superficial mucosa positive for cancer were not stained in some cases of malignant biliary stenosis as a result of study of Tanaka *et al.* [7] on gastric cancer and

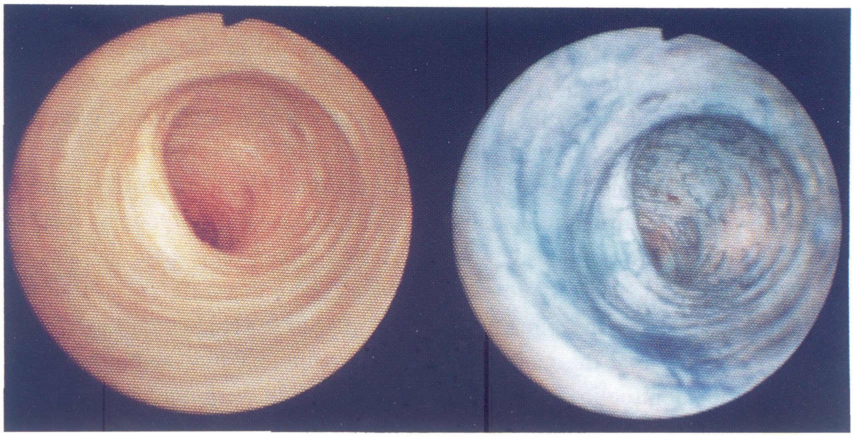

FIGURE 3 Endoscopic views of the common bile duct after complete removal of a common bile duct stone. A. Normal cholangial mucosa with a smooth surface. B. Cholangial mucosa stained with methylene blue.

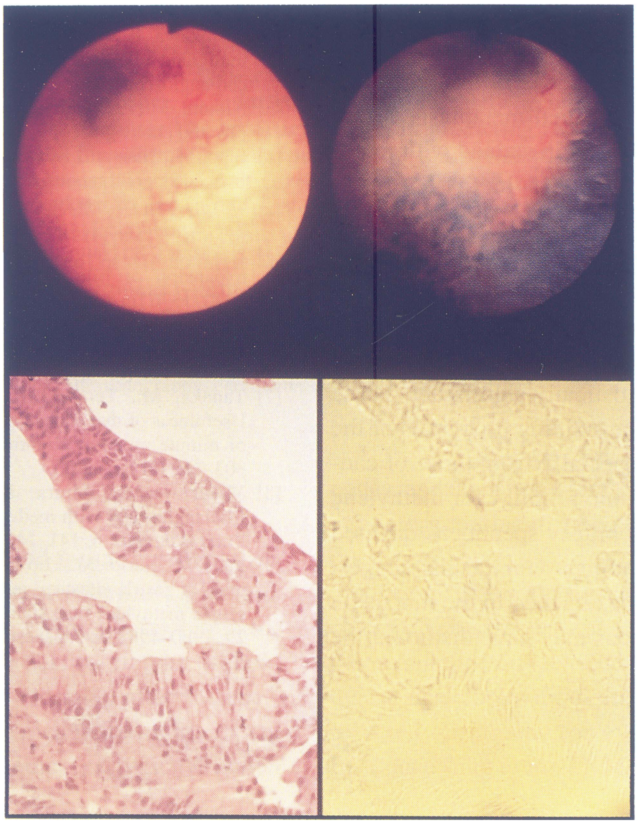

FIGURE 4 Endoscopic views and micrographs of bile duct cancer with mucosal superficial spread. Left top: Papillogranular surface extending from the bile duct stenosis to the hepatic hilum. Right top: The methylene blue staining method identifies the boundary between the area covered with cancerous epithelia and that covered with normal epithelia. Left bottom: Section stained with HE showing papillogranular proliferation with cancerous epithelia. Right bottom: Same section as left bottom: showing lack of methylene blue staining of cancerous epithesought to confirm whether methylene blue just adhered to the superficial mucosa or was absorbed. We therefore studied the specific methylene blue staining properties of the bile duct mucosa, including the normal, metaplastic, and cancerous epithelia. Tada *et al.* [9] studied the methylene blue staining properties of the gastric metaplastic epithelia using frozen sections of the biopsy specimens and demonstrated that methylene blue was absorbed. We used the same method of examination in this study. Because Tada *et al.* [9] reported that the methylene blue absorbed in the superficial epithelia disappeared with time, we placed biopsy specimens in OCT compound, made frozen sections, and immediately observed the specimens microscopically after taking them from the bile duct. Methylene blue pigment was lodged in the epithelial cells of the bile duct, as observed by Tada *et al.* in studying the intestinal metaplastic epithelia of the stomach. However, whether the methylene blue pigment was present in the cytoplasm or the nucleus was not determined. Tada *et al.* reported that intestinal metaplasia with a brush border on the apical portion stained with methylene blue. The mechanism of methylene blue absorption was not described. We have shown that most of the normal epithelia stained well, while the cancerous epithelia did not stain with methylene blue. The number of stained specimens of metaplastic epithelia was less than that of normal epithelia, but the difference was not statistically significant. These results demonstrate that the staining properties of the normal epithelia differ, especially from those of cancerous epithelia. Microscopic findings for methylene blue staining in the seven biopsy specimens differed from their endoscopic findings. Our results suggest that methylene blue may be attached to the mucus on the epithelium, or the mucus may have disturbed the contact of methylene blue with the superficial epithelia. Removal of the superficial mucus will therefore help to accurately evaluate the methylene blue staining properties of cholangial epithelia. This problem remains to be resolved.

In conclusion, our results indicate that the staining of the cholangial epithelia by methylene blue observed during PTCS represents the absorption of methylene blue. There is a difference in methylene blue staining properties between cancerous and normal epithelia. The methylene blue staining method could be helpful in clarifying the boundary of superficial lateral spread of bile duct cancer.

### References

[1] Yamase, H., Nimura, H., Hayakawa, N., *et al.* (1988). Differential diagnosis of stenosis of distal bile duct by percutaneous transhepatic cholangioscopy (PTCS), *Gastroenterol Endosc,* **30**, 1175–1182 [in Japanese].

[2] Nishikawa, K., Ogawa, S., Sato, M., *et al.* (1991). A study of malignant bile duct stenosis using percutaneous transhepatic cholangioscopy, *Dig Endosc,* **3**, 342–349.

[3] Nimura, Y. (1993). Staging of biliary carcinoma: cholangiography and cholangioscopy, *Endoscopy,* **25**, 76–80.

[4] Sato, M., Maetani, I., Ohashi, S., *et al.* (1994). Relationship between percutaneous transhepatic cholangioscopy findings and pattern of carcinomatous spread in the bile duct, *Diag Therap Endosc,* **1**, 45–50.

[5] Nakazawa, S., Naito, T., Ichikawa, M., *et al.* (1978). Epithelial expansion of bile duct carcinoma, *J Gastroenterol,* **75**, 1370–1376 [in Japanese].

[6] Shimada, H., Nimoto, S., Nakagawara, G., *et al.* (1985). The infiltration of bile duct carcinoma along the wall, *J Jpn Surg,* **86**, 179–186 [in Japanese].

[7] Tanaka, M., Terasaki, T., Matsuzaki, K., *et al.* (1990). Usefulness of dye endoscopy and its problems in the diagnosis of minute mucosal abnormalities of the gut, *Endosc Dig,* **2**, 303–312 [in Japanese].

[8] Miyakawa, S., Miura, K., Kawase, K., *et al.* (1985). Cholangioscopy with methylene blue dyeing method, *J Biliary Tract Pancreas,* **6**, 141–147 [in Japanese].

[9] Tada, M., Karita, M., Hirota, K., *et al.* (1987). Endoscopic diagnosis of gastric intestinal metaplasia—reevaluation of the endoscopic methylene blue staining method, *Gastroenterol Endosc,* **29**, 1980–1988 [in Japanese].

